# Dietary Betaine Mitigates Hepatic Steatosis and Inflammation Induced by a High-Fat-Diet by Modulating the Sirt1/Srebp-1/Pparɑ Pathway in Juvenile Black Seabream (*Acanthopagrus schlegelii*)

**DOI:** 10.3389/fimmu.2021.694720

**Published:** 2021-06-23

**Authors:** Min Jin, Yuedong Shen, Tingting Pan, Tingting Zhu, Xuejiao Li, Fangmin Xu, Mónica B. Betancor, Lefei Jiao, Douglas R. Tocher, Qicun Zhou

**Affiliations:** ^1^ Laboratory of Fish and Shellfish Nutrition, School of Marine Sciences, Ningbo University, Ningbo, China; ^2^ Institute of Aquaculture, Faculty of Natural Sciences, University of Stirling, Stirling, United Kingdom; ^3^ Guangdong Provincial Key Laboratory of Marine Biotechnology, Institute of Marine Sciences, Shantou University, Shantou, China

**Keywords:** betaine, hepatic steatosis, high-fat diet, inflammation response, Sirt1/Srebp-1/Pparɑ

## Abstract

The present study aimed to elucidate the mechanism of dietary betaine, as a lipid-lowering substance, on the regulation of lipid metabolism and inflammation in juvenile black seabream (*Acanthopagrus schlegelii*) fed a high fat diet. An 8-week feeding trial was conducted in black seabream with an initial weight of 8.39 ± 0.01g fed four isonitrogenous diets including Control, medium-fat diet (11%); HFD, high-fat diet (17%); and HFD supplemented with two levels (10 and 20 g/kg) of betaine, HFD+B1 and HFD+B2, respectively. SGR and FE in fish fed HFD+B2 were significantly higher than in fish fed HFD. Liver histology revealed that vacuolar fat droplets were smaller and fewer in bream fed HFD supplemented with betaine compared to fish fed HFD. Betaine promoted the mRNA and protein expression levels of silent information regulator 1 (Sirt1), up-regulated mRNA expression and protein content of lipid peroxisome proliferator-activated receptor alpha (*pparα*), and down-regulated mRNA expression and protein content of sterol regulatory element-binding protein-1(*srebp-1*). Furthermore, the mRNA expression levels of anti-inflammatory cytokines in liver and intestine were up-regulated, while nuclear factor kB *(nf-kb*) and pro-inflammatory cytokines were down-regulated by dietary betaine supplementation. Likewise, in fish that received lipopolysaccharide (LPS) to stimulate inflammatory responses, the expression levels of mRNAs of anti-inflammatory cytokines in liver, intestine and kidney were up-regulated in fish fed HFD supplemented with betaine compared with fish fed HFD, while *nf-kb* and pro-inflammatory cytokines were down-regulated. This is the first report to suggest that dietary betaine could be an effective feed additive to alleviate hepatic steatosis and attenuate inflammatory responses in black seabream fed a high fat diet by modulating the Sirt1/Srebp-1/Pparɑ pathway.

## Introduction

High-fat diets have been used widely in aquaculture as they can spare dietary protein, promote growth performance, and reduce nitrogen and phosphorus emissions (1, 2). However, high-fat diets can also cause lipid accumulation and metabolic disorders, endoplasmic reticulum stress (ERS), inflammation, and lipidic toxicity in fish (3–7). Recently, several studies have evaluated lipid-lowering substances such as L-carnitine, choline and betaine in fish including common allogynogenetic gibel carp (*Carassius auratus gibelio*) (8), common carp (*Cyprinus carpio*) (9), black seabream (*Acanthopagrus schlegelii*) (5, 6) and blunt snout bream (*Megalobrama amblycephala*) (10, 11). While an appropriate feed additive to protect against pathologies induced by high-fat diets is urgently required, the mechanisms underlying how feed additives attenuate lipid accumulation, inflammatory responses and ERS remains to be elucidated.

Betaine is a methyl derivative of glycine, first discovered as a natural byproduct in sugar beets (*Beta vulgaris*) and then shown to have widespread distribution in many plants, microorganisms, marine invertebrates, and animals (12). Betaine is also an important nutrient in aquatic feed as it can have several beneficial impacts, such as improved feed intake, growth performance and survival, as reported in various fish species (13–15). In recent years, research has focused on the possible mechanisms by which betaine can regulate lipid metabolism, reduce fat accumulation and, consequently, alleviate hepatic steatosis. Typically, betaine has been used widely in mammals to treat fatty liver disease (12, 16–21). Similar effects have been reported in fish species, with dietary betaine supplementation shown to reduce lipid deposition by modulating the expression of lipid metabolism genes (8, 11). Furthermore, the role of betaine as a methyl donor in one-carbon metabolism in liver has been of renewed interest for reducing liver injury (12, 21). In addition, studies have also shown that betaine can modulate oxidative stress and inflammation, as well as improve immune function by increasing antioxidant enzymes (e.g. superoxide dismutase, glutathione peroxidase, glutathione transferase), promoting expression of anti-inflammatory cytokines, suppressing nuclear factor kB (NFκB) activation and, consequently, decreasing pro-inflammatory cytokine expression (20, 22–26). Therefore, betaine is a promising candidate additive for aquatic feed to mitigate lipid accumulation and inflammation induced by feeding high-fat diets.

Sirtuin 1 (SIRT1) is a highly conserved NAD^+^-dependent protein deacetylase, recognized as a master switch in energy homeostasis, which plays an important role in cellular metabolic processes including stress responses and inflammation (27, 28). Rodgers and Puigserver (27) reported that hepatic SIRT1 is an important factor in the regulation of lipid metabolism that itself is regulated by transcription factors including sterol regulatory element-binding proteins (SREBPs), liver X receptor (LXR) and peroxisome proliferator activated receptors (PPARs). In mammals, studies showed that low levels of serum SIRT1 were observed in obese patients with liver steatosis, and liver-specific SIRT1-knockout mice showed hepatic lipid accumulation, suggesting that SIRT1 was a negative modulator of the SREBP-1 lipogenic pathway (28–31). Meanwhile, as a vital pathway for the regulation of hepatic lipid metabolism, SIRT1 positively regulates PPARα to reduce lipid accumulation in liver (27). Furthermore, in mammals, SIRT1 could suppress NF-kB signaling that, in turn, attenuated NF-kB driven inflammation (32, 33). Accumulating evidence confirmed that PPARα regulated metabolism and inflammation *via* SIRT1 (34). However, the metabolic regulatory functions of betaine in reducing lipid accumulation and inflammation caused by high-fat diets in fish, and whether betaine functions *via* modulating the Sirt1/Srebp-1/Pparɑ pathway has not been demonstrated.

Black seabream is an important marine fish species that is cultured commercially on the southeast coast of China, Japan, South Korea and other countries in Southeast Asia, and is regarded as an excellent species for intensive aquaculture as it exhibits rapid growth, high disease resistance, and can tolerate a wide range of environmental conditions as described previously (5, 6). Moreover, black seabream is a validated experimental model for high-fat diet-induced inflammation in fish as confirmed in our previous studies (5, 6). Therefore, the present study aimed to investigate whether dietary betaine can ameliorate high-fat diet-induced hepatic lipid accumulation and inflammation response modulation of the Sirt1/Srebp-1/Pparɑ pathway in black seabream, and provide further insight into the mechanism of action of dietary betaine in fish.

## Materials and Methods

### Experimental Design and Diet Preparation

Four isonitrogenous (41% crude protein) experimental diets with two levels of lipid (11% and 17% crude lipid) were formulated with the diets containing the higher lipid level supplemented with two levels of betaine (Sinopharm Chemical Reagent Co., Ltd., Shanghai, China). The diets were termed as (1) Control, medium-fat diet (11%), (2) HFD, high-fat diet (17%), (3) HFD+B1, HFD with betaine supplement (10 g/kg dry diet), (4) HFD+B2, HFD with betaine supplement (20 g/kg dry diet) (**Table 1**). Fishmeal, soybean protein concentrate and soybean meal were used as protein sources, with fish oil, palmitic acid and soybean lecithin used as the main lipid sources. All ingredients were purchased from Ningbo Tech-Bank Feed Co. Ltd., Ningbo, China. The experimental diets were produced according to the methods described in detail previously (5). Briefly, the ground ingredients were mixed in a Hobart type mixer and cold-extruded pellets produced (F-26, Machine factory of South China University of Technology) with pellet strands cut into uniform sizes (2 mm and 4 mm diameter pellets) (G-250, Machine factory of South China University of Technology). Pellets were heated for 30 min at 90°C, and then air-dried to approximately 10% moisture, sealed in vacuum-packed bags and stored at −20°C until used in the feeding trial.

**Table 1:** Formulation and composition of the experimental diets (% dry matter).

Ingredient	Diets			
	Control	HFD	HFD+B1	HFD+B2
Fish meal	26.00	26.00	26.00	26.00
Soybean protein concentrate	10.00	10.00	10.00	10.00
Soybean meal	20.00	20.00	20.00	20.00
Wheat flour	23.30	23.30	23.30	23.30
Fish oil	8.00	8.00	8.00	8.00
Palmitic acid	0.00	6.00	6.00	6.00
Soybean lecithin	1.00	1.00	1.00	1.00
Vitamin premix^a^	0.50	0.50	0.50	0.50
Mineral premix^b^	2.00	2.00	2.00	2.00
Betaine	0.00	0.00	1.00	2.00
Choline chloride	0.20	0.20	0.20	0.20
Ca(H_2_PO_4_)_2_	1.00	1.00	1.00	1.00
Cellulose	8.00	2.00	1.00	0.00
*Proximate composition (%)*				
Dry matter	89.34	89.96	89.85	88.61
Crude protein	40.69	41.16	41.99	41.71
Crude lipid	10.89	16.60	16.97	16.52
Ash	9.35	9.40	9.32	9.25

a Vitamin premix based on Zhou et al. (35).

b Mineral mixture (g kg^−1^ premix): FeC_6_H_5_O_7_, 11.43; ZnSO_4_·7H_2_O, 11.79; MnSO_4_·H2O (99%), 2.49; CuSO_4_·5H_2_O (99%), 1.06; MgSO_4_·7H_2_O (99%), 27.31; KH_2_PO_4_, 233.2; NaH_2_PO_4_, 228.39; C_6_H_10_CaO_6_·5H_2_O (98%), 34.09; CoC_l2_·6H_2_O (99%), 0.54. KIO_3_ (99%), 0.06; zeolite, 449.66.

### Feeding Trial and Experimental Conditions

Black seabream juveniles (initial weight 8.39 ± 0.01 g) were obtained from a local commercial hatchery at Xiangshan Bay, Ningbo, China. Prior to the start of the experiment, black seabream juveniles were acclimated to the experimental facilities and fed a commercial diet (45% protein, 12% crude lipid, Ningbo Tech-Bank Corp.) for two weeks. The feeding trial was carried out with a completely randomized design. A total of 360 black seabream juveniles were randomly allocated to 12 floating net cages (1.5 m × 1.5 m × 2.0 m) corresponding to triplicate cages for each of the four dietary treatments. Fish were hand-fed to apparent satiation twice daily at 07:00 and 17:00 over eight weeks. During the experimental period, seawater conditions including temperature (26.6 - 30.7°C), salinity (22.53 - 27.86 g/L), dissolved oxygen (4.7 - 6.8 mg/L) and pH (8.0 - 8.1 mg/L) were measured with YSI Proplus (YSI, Yellow Springs, Ohio, USA).

### Sample Collection

At the end of feeding trial, fish were sampled 24 h after the last feed. All fish were euthanized (MS-222 at 10 mg/L) and all fish in each cage were individually weighed and counted to determine specific growth rate (SGR) and calculate feed efficiency (FE). The experimental replicates (units) were cages and so all analyses were performed on a per cage basis (n = 3) with samples from each cage being derived from pooled fish. Dorsal muscle and liver samples (pools of 3 fish per cage) were collected and stored at -80˚C for analyzing proximate composition. Liver, and intestine samples were also collected and stored at -80˚C until further analysis of biochemical indices (pools of 3 fish per cage), gene expression (pools of 3 fish per cage) and protein determination (pools of 2 fish per cage). Fresh liver tissues were collected into 4% formaldehyde from one fish per cage for histological analyses. Blood samples were taken from the caudal vasculature of 8 fish per cage using 2ml syringes, pooled, and stored at 4°C for 24 h for serum biochemical indices.

### Proximate Composition Analysis

Lipid, protein, moisture and ash contents of feed, liver and dorsal muscle samples were determined by standard AOAC methods (36). Moisture content was measured by drying the samples to constant weight at 105°C. Total lipid (crude lipid in the case of feed) was extracted *via* the ether extraction method using a Soxtec System HT (Soxtec System HT6, Tecator, Sweden). Protein (crude protein in the case of feed) (N × 6.25) was determined according to the Dumas combustion method with a protein analyzer (FP-528, Leco, USA) and ash content was measured using a muffle furnace at 550°C for 8 h.

### Assay of Serum and Liver Biochemical Indices

Serum was collected from blood by centrifugation at 956 g for 10 min at 4°C. Triacylglycerol and cholesterol contents in serum were assayed using an automatic biochemistry analyzer (VITALAB SELECTRA Junior Pros, Netherlands). Liver samples were homogenized in nine volumes (w/v) of ice-cold physiological saline 0.89% (w/v), and then centrifuged as above. The contents of adiponectin (ADP), S-adenosylmethionine (SAM), S-adenosylhomocysteine (SAH) and homocysteine (Hcy) (Nanjing Jiancheng Bioengineering Institute, China), as well as triacylglycerol and cholesterol (Shanghai Qiaodu biotechnology Co., Ltd, China) were assayed using commercial kits according to the manufacturer’s instructions by Multiskan spectrum (Thermo, USA).

### Histological Analysis of Liver

Fresh liver tissue was fixed in 4% paraformaldehyde before paraffin sections were prepared (Servicebio, Hangzhou, China). Briefly, after fixation for at least 24 h, tissue samples were trimmed appropriately in a fume hood before being dehydrated in ethanol with concentration increasing incrementally from 75% to 100%. Liver samples were then embedded in paraffin, sliced into sections of 4 μm using a microtome, and stained with haematoxylin and eosin (H&E), and images acquired under a microscope (Nikon Eclipse CI, Tokyo, Japan).

### Total RNA Extraction, Reverse Transcription and Real-time PCR

Total RNA of liver and intestine were extracted by the TRIzol method, and quality and quantity assessed by 1.0% agarose gel electrophoresis and spectrophotometer NanoDrop 2000 (Thermo Fisher Scientific, USA). The cDNA was prepared from 1000 ng of DNAase-treated RNA and synthesized using PrimeScript™ RT Reagent Kit with gDNA Eraser (Perfect Real Time) (Takara). The housekeeping gene *β-actin* was used as reference gene after confirming its stability across the experimental treatments. Specific primers for the candidate genes *pparα*, *cpt1a*, *lpl*, *accα*, *fas*, *srebp-1*, *tnf-α*, *il-1β*, *nf-kb*, *tgfβ-1*, *il-10* and *sirt1* used for qPCR were designed by Primer Premier 5.0 (**Table 2**). Amplification was performed using a quantitative thermal cycler (Lightcycler 96, Roche, Switzerland). The qPCR assays were performed in a total volume of 20 μL, containing 0.4 μL of each primer, 10 μL of 2×ChamQ SYBR qPCR Green Master Mix (Vazyme), 0.8 μL of 1/8 diluted cDNA and 8.4 μL DEPC-water. The thermal-cycling conditions used for qPCR were as follows: 95°C for 2 min, followed by 45 cycles of 95°C for 10 s, 58°C for 10 s and 72°C for 20 s. Standard curves were generated using six different dilutions (in triplicate) of the cDNA samples, and the amplification efficiency was analyzed using the equation E=10^(–1/Slope)^-1. The amplification efficiencies of all genes were approximately equal and ranged from 87 to 109%. Data were presented as relative gene expression with regards to the expression values in fish fed the Control diet (reference group). The expression levels of the target genes were calculated using the 2^–ΔΔCt^ method as described by Livak and Schmittgen (39).

**Table 2:** Primers for real-time quantitative PCR.

Gene	Nucleotide sequence (5’ –3’)	Size (bp)	GenBank reference or Publication	Functions
*il-1β^1^*	F: CATCTGGAGGCGGTGAA	231	JQ973887	Pro-inflammation cytokine
	R: CGGTTTTGGTGGGAGGA			
*tnf-α^2^*	F: GTCCTGCTGTTTGCTTGG	154	AY335443	Pro-inflammation cytokine
	R: AATGGATGGCTGCCTTGG			
*nf-κb^3^*	F: AGCCCAAGGCACTCTAGACA	154	MK922543	Nuclear transcription factor
	R: GTTCTGGGCAGCTGTAGAGG			
*tgfβ-1^4^*	F: GGGTTTCCAACTTCGGC	209	Xue et al. (37)	Anti-inflammation cytokine
	R: TTGTGTCCGTGGAGCGT			
*il-10^5^*	F:TGTCAAACGGTTCCTTGCAG	172	MK922542	Anti-inflammation cytokine
	R: GGCATCCTGGGCTTCTATCT			
*accα* ^6^	F: AGTAGCCTGATTCGTTGGT	154	KX066238	Lipogenesis pathway
	R: AGTAGCCTGATTCGTTGGT			
*fas* ^7^	F: AAGAGCAGGGAGTGTTCGC	213	KX066240	Lipogenesis pathway
	R: TGACGTGGTATTCAGCCGA			
*srebp*-*1* ^8^	F: TGGGGGTAGGAGTGAGTAG	247	KX066235	Lipogenesis pathway
	R: GTGAAGGGTCAGTGTTGGA			
*lpl* ^9^	F: CTGCTACTCCTCTGCCCA	204	KX078571	Lipolysis pathway
	R: ACATCCCTGTTACCGTCC			
*cptla* ^10^	F: TGCTCCTACACACTATTCCCA	203	KX078572	Lipolysis pathway
	R: CATCTGCTGCTCTATCTCCCG			
*pparα* ^11^	F: ACGACGCTTTCCTCTTCCC	183	KX066234	Lipolysis pathway
	R: GCCTCCCCCTGGTTTATTC			
*sirt* ^12^	F: TGGATGAAACTGTAGGAACC	238	MN871952	metabolic sensor
	R: ACAACAATGGACTGGGAA			
*β-actin*	F: ACCCAGATCATGTTCGAGACC	212	Jiao et al. (38)	Housekeeping gene
	R: ATGAGGTAGTCTGTGAGGTCG			

^1^il-1β, interleukin 1 beta; ^2^ tnf-α, tumor necrosis factor alpha; ^3^ nf-κb, nuclear factor-kappa B; ^4^ tgfβ-1, transforming growth factor beta-1; ^5^ il-10, interleukin-10; ^6^ accα, acetyl-CoA carboxylase alpha; ^7^ fas, fatty acid synthase; ^8^ srebp-1, sterol regulator element-binding protein-1; ^9^ lpl, lipoprotein lipase; ^10^ cpt1a, carnitine palmitoyltransferase 1A; ^11^ pparα, peroxisome proliferators-activated receptor alpha; ^12^ sirt1, Silent information regulator 1.

### Lipopolysaccharide Injection and Sampling

After the 8-week feeding trial, six fish in each cage were randomly collected for LPS challenge to intensify inflammatory responses. LPS (Escherichia coli 055: B5; Sigma-Aldrich, USA) was dissolved in sterile phosphate-buffered saline (PBS, pH = 7.4) to a final concentration of 0.5 mg/ml. Three fish from the HFD, HFD+B1 and HFD+B2 treatments were individually injected intraperitoneally with 0.2 ml LPS at a dose of 2.5 mg/kg body weight. As control, the remaining three fish per treatment were injected individually with the same volume of sterile PBS. Liver, intestine and kidney were collected from all fish 24 h after injection (3 fish pooled with LPS per cage, 3 fish pooled with PBS per cage, n = 3 per dietary treatment), and snap frozen in liquid nitrogen and stored at -80°C for later gene expression analysis of *nf-kb*, *il-1β*, *tnf-α*, *tgfβ-1*and *il-10*.

### Protein Extraction and Western Blot Analysis

Total protein was extracted from liver tissues using RIPA lysis buffer according to the manufacturer’s instructions (Solarbio, China), and protein concentrations determined using a BCA Protein Assay kit (Nanjing Jiancheng, China) according to the manufacturer’s instructions. Protein samples were resolved by sodium dodecyl sulfate-polyacrylamide gel electrophoresis (SDS-PAGE) at a constant voltage of 100 V for 1 h. Proteins were then transferred onto a 0.45 µm PVDF membrane and subsequently blocked with 5% skimmed milk in TBST for 2 h. The membrane was rinsed briefly with TBST prior to probing with primary antibodies: anti-β-actin (Abclonal, 1:1000 dilution), anti-Sirt 1 (Cell Signaling Technology, USA, 1:200 dilution), at 4°C overnight. The PVDF membrane was then washed and incubated with secondary antibody (Abclonal, China, 1:2000 dilution) for 1 h at room temperature and immunoreactive bands visualized with a luminescent image analyzer and quantified by Image J 1.52a (Tanon 5200, China).

### Determination of Srebp-1 and Pparα Proteins in Liver

Liver samples were homogenized in nine volumes (w/v) of ice-cold phosphate buffer saline (PBS, pH 7.4) (w/v), then centrifuged at 664 x g for 20 min at 4°C, and the supernatant collected for further assays. The protein concentrations of Srebp-1 and Pparα were assayed using commercial ELISA kits for fish (Shanghai Enzyme-linked Biotechnology Co., Ltd., Shanghai, China) according to the manufacturer’s instructions using Multiskan spectrum (Thermo, USA).

### Statistical Analysis

Results are presented as means and SEM (number of replicates as indicated). The relative gene expression results (qPCR analyses) were expressed as mean normalized ratios corresponding to the ratio between the copy number of the target gene and the copy number of the reference gene, *β-actin*. The homogeneity of variances (Levene’s test) were checked prior to one-way analysis of variance (ANOVA) followed by Tukey’s HSD test at a significance level of *P* ≤ 0.05 (IBM SPSS Statistics 20, USA).

## Results

### Growth Performance, Feed Utilization, Survival and Proximate Composition

**Figure 1:**
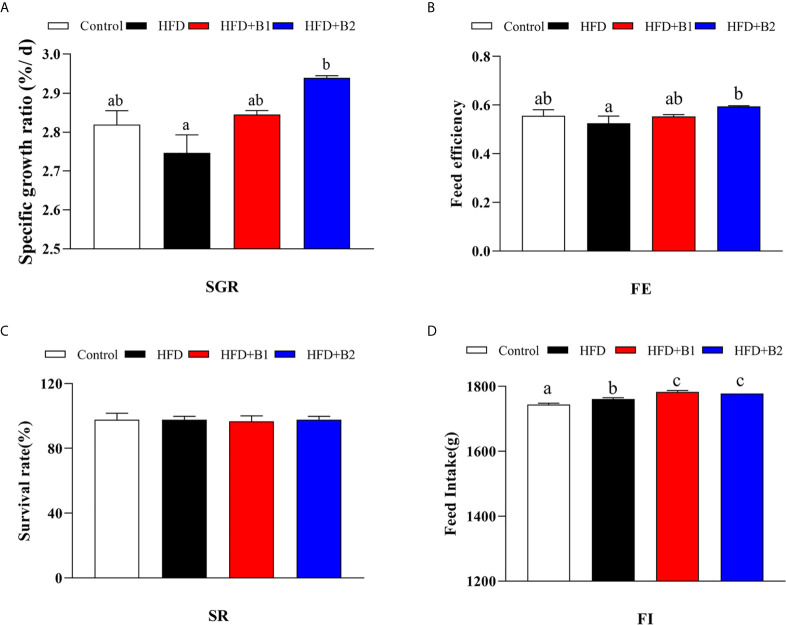
Growth performance and feed utilization of juvenile black seabream (*Acanthopagrus schlegelii*) fed the experimental diets (Control: 11% crude lipid; HFD: high-fat diet; HFD+B1: HFD +betaine (10g/kg); HFD+B2: HFD + betaine (20 g/kg)) for 8 weeks. **(A)** Specific growth ratio (SGR): SGR (%/d) = 100 × (Ln (final body weight)- Ln (initial body weight))/56 days. **(B)** Feed efficiency (FE): FE = weight gain (g, wet weight)/feed consumed (g, dry weight). **(C)** Survival rate (SR): SR (%) = 100 (final fish number/initial fish number). **(D)** Total dry feed intake (FI) (during the feeding trial of 8 week). Values are means (n=3) with standard errors represented by vertical bars. Mean values for the same column with different letters were significantly different (*P* < 0.05).

**Figure 2:**
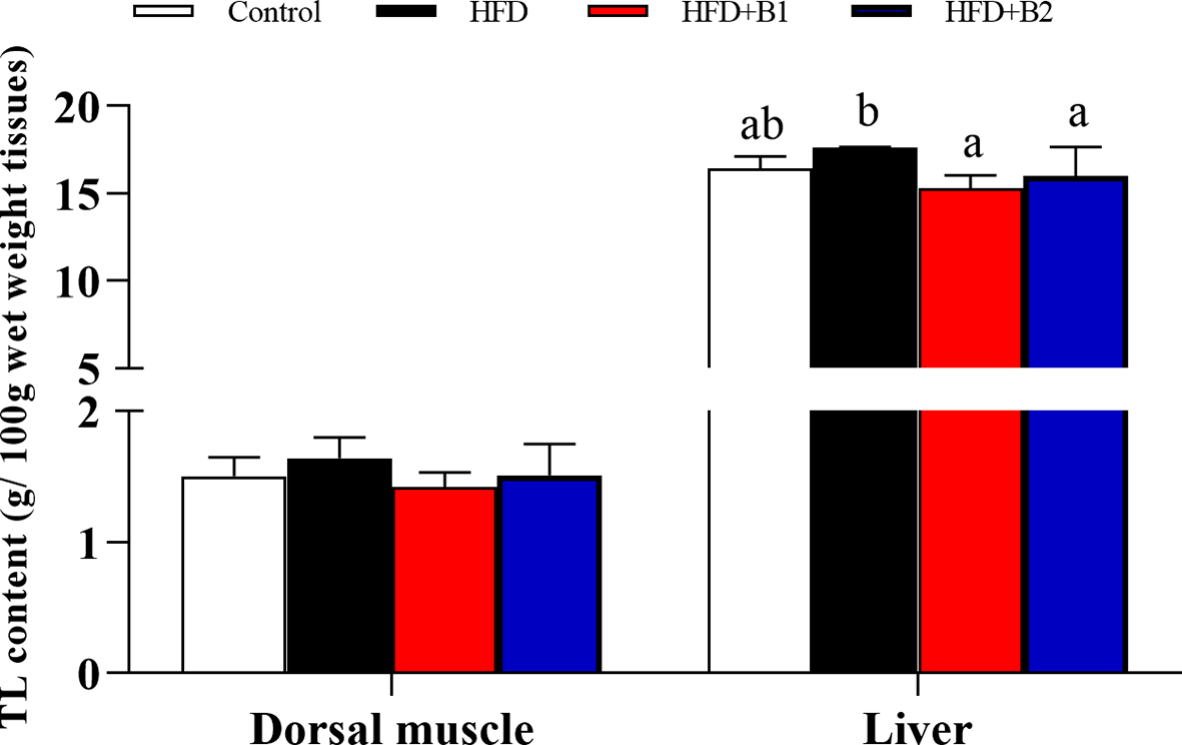
Dorsal muscle and liver lipid contents of juvenile black seabream (*Acanthopagrus schlegelii*) (g/100g wet weight tissues) fed the experimental diets (Control: 11% crude lipid; HFD: high-fat diet; HFD+B1: HFD +betaine (10g/kg); HFD+B2: HFD + betaine (20 g/kg)) for 8 weeks. TL:total lipid. Values are means (n=3), with standard errors represented by vertical bars. Mean values for the same column with different letters were significantly different (*P* < 0.05).

Highest specific growth rate (SGR) and feed efficiency (FE) were recorded in fish fed HFD-B2, significantly higher than fish fed HFD (**Figures 1A, B**). Survival rate (SR) did not show any significant differences among treatments (**Figure 1C**). Fish fed diets containing betaine (HFD+B1and HFD+B2) showed significantly higher feed intake (FI) than fish fed the other diets (**Figure 1D**). Fish fed the HFD diets supplemented with betaine had significantly lower hepatic lipid contents compared to fish fed HFD. Similar results were found for dorsal muscle lipid content that showed a decreasing trend with dietary betaine supplementation, albeit not significantly different to HFD (**Figure 2**).

### Serum and Hepatic Biochemical Indices

**Figure 3:**
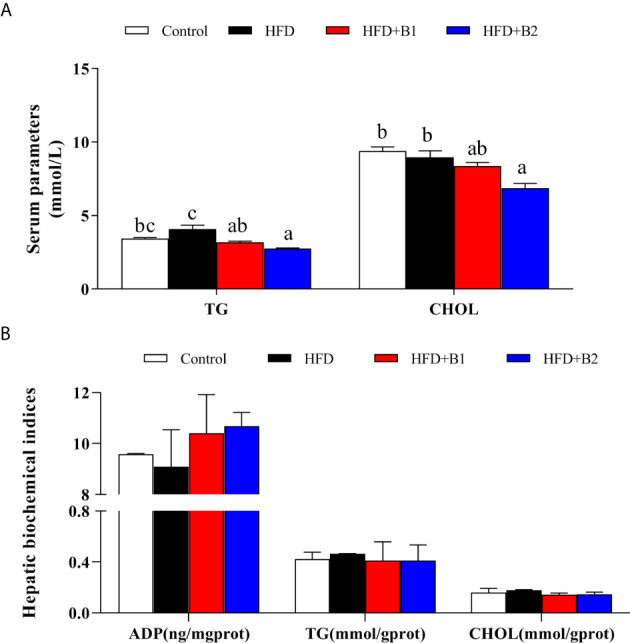
Serum and hepatic parameters of juvenile black seabream (*Acanthopagrus schlegelii*) (% wet weight) fed the experimental diets (Control: 11% crude lipid; HFD: high-fat diet; HFD+B1: HFD +betaine (10g/kg); HFD+B2: HFD + betaine (20 g/kg)) for 8 weeks. **(A)** Serum triacylglycerol (TG) and cholesterol (CHOL) contents. **(B)** hepatic adiponectin (ADP), TG and CHOL contents. Values are means (n=3) with standard errors represented by vertical bars. Mean values for the same column with different letters were significantly different (*P* < 0.05).

Fish fed HFD+B2 had significantly lower serum concentrations of triacylglycerol and cholesterol compared to fish fed the HFD diet (**Figure 3A**). In contrast, no significant differences were found among treatments in hepatic contents of triacylglycerol or cholesterol. Likewise, similar results were found for hepatic ADP as there was a clear trend for dietary betaine to increase ADP compared with fish fed HFD, although no statistical differences were found (**Figure 3B**).

### Hepatic Histological Analysis

**Figure 4:**
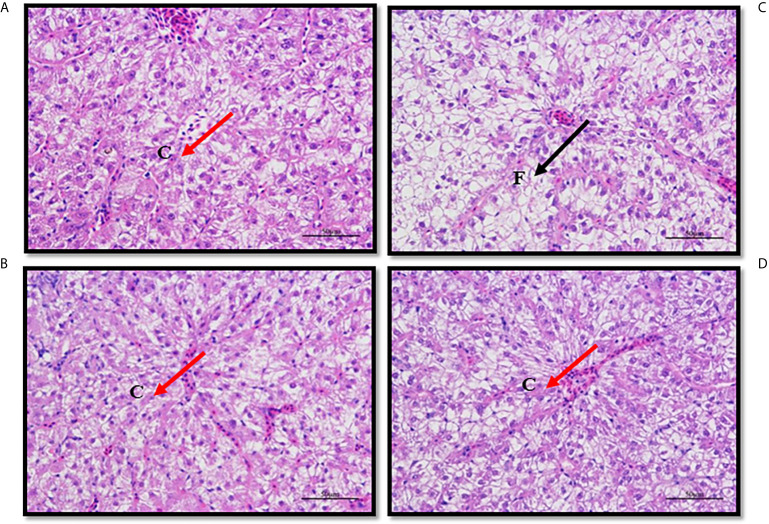
Paraffin section of liver of juvenile black seabream (*Acanthopagrus schlegelii*) fed the experimental diets (Control: 11% crude lipid; HFD: high-fat diet; HFD+B1: HFD +betaine (10g/kg); HFD+B2: HFD + betaine (20 g/kg)) for 8 weeks. The liver sections were stained with H&E to enhance the contrast (400X). **(A)** Paraffin section of liver of Control group; **(B)** Paraffin section of liver of HFD group; **(C)** Paraffin section of liver of HFD+B1 group; **(D)** Paraffin section of liver of HFD+B2 group. F, Fat drop; C, Cell nucleus.

In fish fed the Control diet (medium fat), hepatocyte shape and structure were regular and normal, the nucleus with nucleolus was spherical and basically in the middle, the arrangement was relatively tight and most of the nuclei were in the middle of the cells (**Figure 4A**). However, in fish fed HFD, the hepatocytes were mostly swollen and nuclei were offset to the edge of the cell, or difficult to distinguish, and many vacuolar lipid droplets of varying size were observed (**Figure 4B**). Compared with fish fed HFD, in fish fed HFD+B1 and HFD+B2 the shape of hepatocytes was more regular and fewer vacuolar lipid droplets were observed (**Figures 4C, D**).

### Hepatic Methionine Metabolic Pathway Key Markers

**Figure 5:**
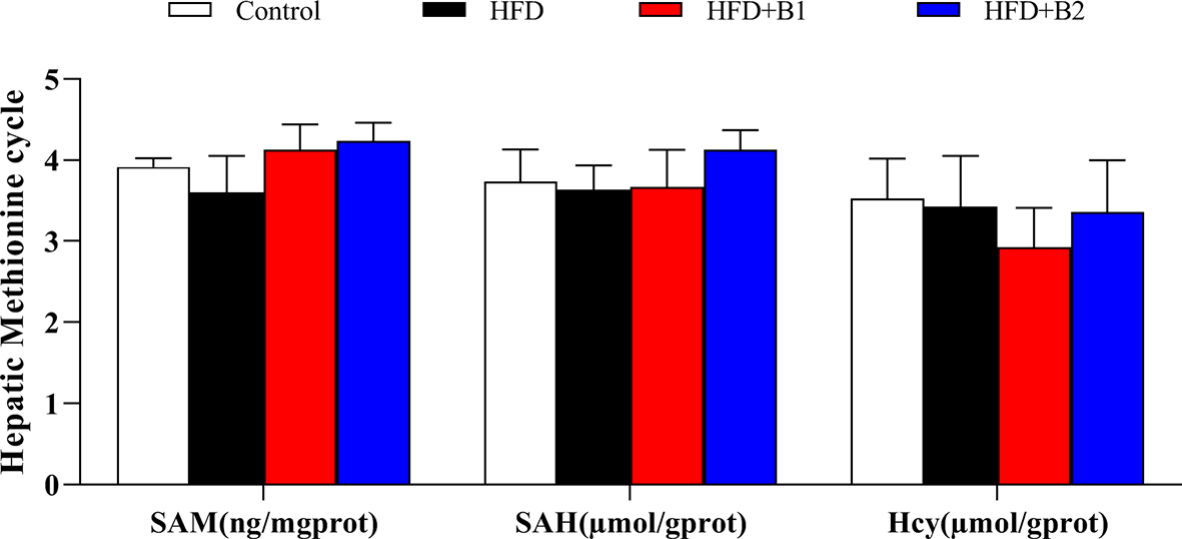
Hepatic methionine metabolic pathway markers of juvenile black seabream (*Acanthopagrus schlegelii*) fed the experimental diets [Control: 11% crude lipid; HFD, high-fat diet; HFD+B1, HFD +betaine (10g/kg); HFD+B2, HFD + betaine (20 g/kg)] for 8 weeks. SAM, S-adenosylmethionine; SAH, S-adenosylhomocysteine; Hcy, homocysteine. Values are means (n=3) with standard errors represented by vertical bars.

As a donor of methyl groups, betaine is an essential biochemical component of the hepatic methionine cycle and, therefore, some key metabolites of the hepatic methionine cycle were measured (**Figure 5**). Hepatic contents of SAM, SAH and Hcy were not significantly different between fish fed HFD or HFD supplemented with betaine. However, although not statistically significant, compared to fish fed HFD, fish fed HFD supplemented with betaine showed numerically lower hepatic Hcy levels and increased hepatic SAM contents.

### Regulatory Factor, Lipolytic and Lipogenic Pathway Key Markers

**Figure 6:**
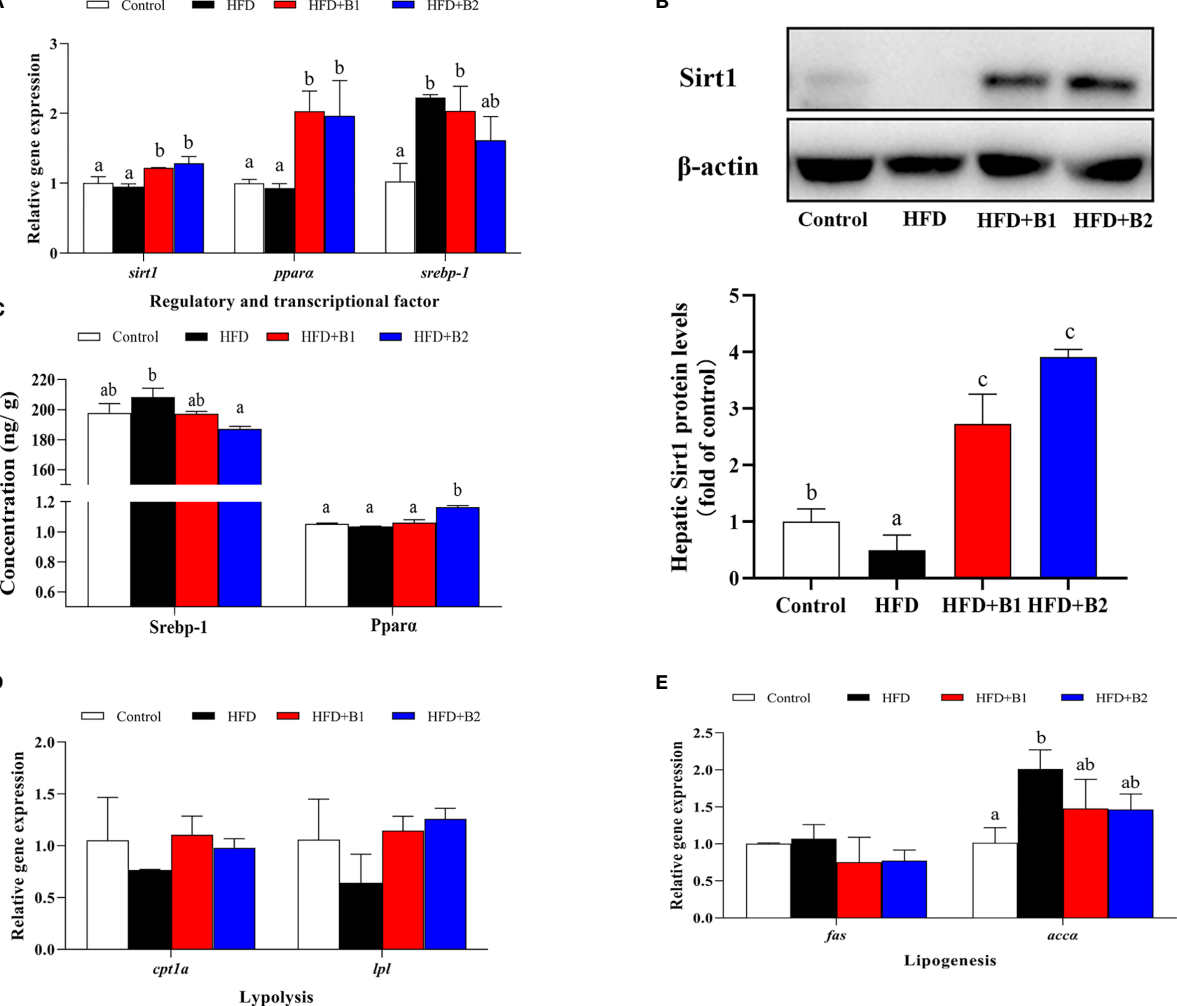
Lipid metabolism gene expression and protein concentration/expression in liver of juvenile black seabream (*Acanthopagrus schlegelii*) (% wet weight) fed the experimental diets [Control: 11% crude lipid; HFD: high-fat diet; HFD+B1: HFD +betaine (10g/kg); HFD+B2: HFD + betaine (20 g/kg)] for 8 weeks. **(A)** The relative mRNA expression of hepatic *sirt1*, *ppara*, and *srebp-1*. **(B)** The hepatic Sirt1 protein level. **(C)** The protein concentration of hepatic SREBP-1 and PPARɑ. **(D)** The relative mRNA expression of hepatic *cpt1a* and *lpl*. **(E)** The relative mRNA expression of hepatic *fas* and *accɑ*. Control was used as the reference group, and the mRNA expression levels of target genes were normalized relative to the expression of *β-actin*. Values are means (n = 3) with standard errors represented by vertical bars. Mean values for the same gene with different letters were significantly different (*P* < 0.05).

In order to determine the mechanism of Sirt1/Srebp-1/Pparɑ pathway-mediated dietary betaine regulation of lipid metabolism, the expression of genes related to several lipid metabolic pathways including regulatory and transcriptional factors, lipolytic pathway and lipogenic pathway were determined in the liver of black seabream juveniles (**Figure 6**). Compared to fish fed HFD, the expression levels of *sirt1* and *pparɑ* genes in liver were significantly up-regulated in fish fed the diets with betaine supplementation, whereas hepatic expression of the *srebp-1* gene was lower, albeit not significantly, in fish fed the HFD+B2 diet (**Figure 6A**). To confirm that the effects on expression of the genes of the pathway were reflected in protein expression, the protein contents of the major components were determined in liver. The expression of Sirt1 in fish fed HFD supplemented with betaine was markedly higher compared to fish fed HFD (**Figure 6B**). Similarly, the protein concentration of Pparɑ in fish fed the HFD+B2 diet was significantly higher than fish fed HFD, while the opposite was the case for Srebp-1 where, compared to fish fed HFD, fish fed the HFD+B2 diet showed lower hepatic Srebp-1 protein concentration (**Figure 6C**). Although generally not statistically different, consistent results were obtained for genes of the lipolytic and lipogenic pathways. Thus, in the lipolytic pathway, hepatic *cpt1a* and *lpl* mRNA expression was numerically higher in fish fed diets supplemented with betaine compared with fish fed HFD (**Figure 6D**). In the lipogenic pathway, similar to the significant effects on *srebp-1*, expression levels of *fas* and *accɑ* showed decreasing trends with dietary betaine supplementation, albeit not significantly different to HFD (**Figure 6E**).

### Inflammatory Markers After 8-week Feeding Trial

**Figure 7:**
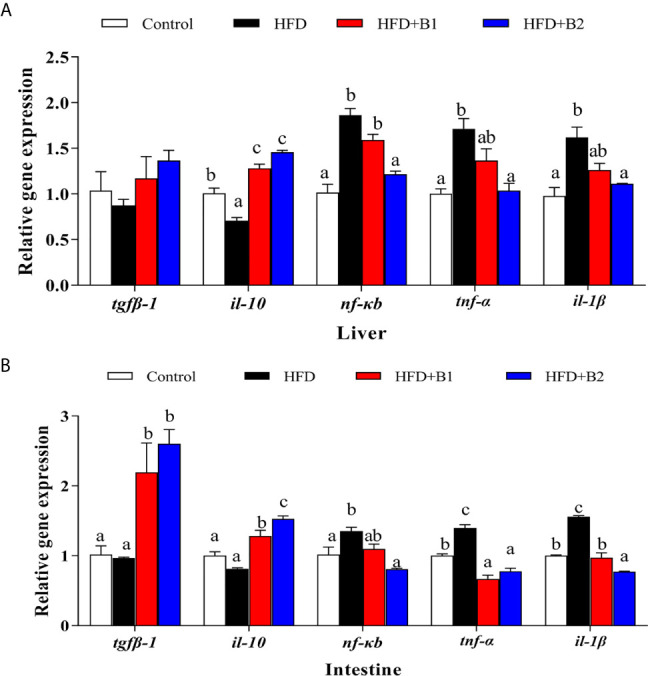
Inflammation gene expression in liver and intestine of juvenile black seabream (*Acanthopagrus schlegelii*) (% wet weight) fed the experimental diets [Control: 11% crude lipid; HFD: high-fat diet; HFD+B1: HFD +betaine (10g/kg); HFD+B2: HFD + betaine (20 g/kg)] for 8 weeks. **(A)** The relative mRNA expression of *tgfβ-1*, *il-10*, *nfκb*, *tnf-α* and *il-1β* in liver. **(B)** The relative mRNA expression of *tgfβ-1*, *il-10*, *nfκb*, *tnf-α* and *il-1β* in intestine. Control was used as the reference group, and the mRNA expression levels of target genes were normalized relative to the expression of *β-actin*. Values are means (n = 3) with standard errors represented by vertical bars. Mean values for the same gene with different letters were significantly different (*P* < 0.05).

The expression levels of genes of the inflammatory response including nuclear transcription factor *nf-kb*, pro-inflammatory cytokines *il-1β* and *tnf-α* as well as anti-inflammatory cytokines *tgfβ-1* and *il-10* in liver and intestine are shown in **Figure 7**. In liver and intestine, the expression levels of *nf-kb*, *tnf-α* and *il-1β* were significantly up-regulated in fish fed HFD compared to fish fed the control (medium fat) diet, and down-regulated in fish fed the diet supplemented with the higher level of betaine (HFD+B2). The expression level of *tgfβ-1* in intestine as well as *il-10* in liver and intestine were significantly up-regulated in fish fed the HFD diets supplemented with betaine.

### Inflammatory Markers After Lipopolysaccharide Injection

**Figure 8:**
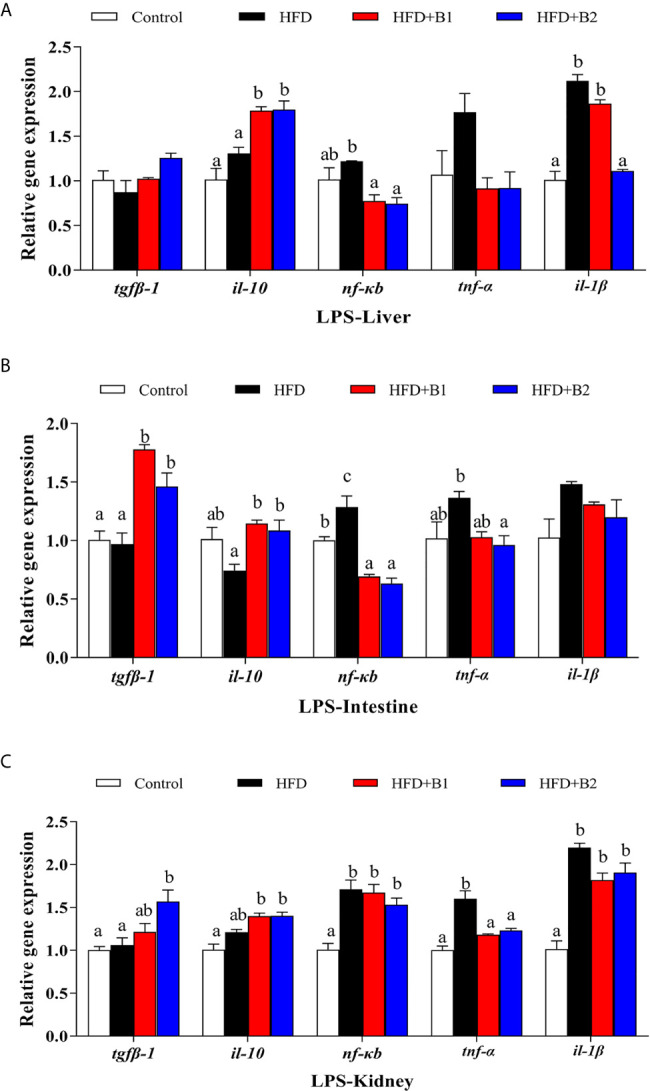
Effects of LPS on inflammation gene expression in liver, intestine and kidney of juvenile black seabream (*Acanthopagrus schlegelii*) (% wet weight) fed the experimental diets [Control: 11% crude lipid; HFD: high-fat diet; HFD+B1: HFD +betaine (10g/kg); HFD+B2: HFD + betaine (20 g/kg)] for 8 weeks. **(A)** The relative mRNA expression of *tgfβ-1*, *il-10*, *nfκb*, *tnf-α* and *il-1β* in liver. **(B)** The relative mRNA expression of *tgfβ-1*, *il-10*, *nfκb*, *tnf-α* and *il-1β* in intestine. **(C)** The relative mRNA expression of *tgfβ-1*, *il-10*, *nfκb*, *tnf-α* and *il-1β* in kidney. Control was used as the reference group, and the mRNA expression levels of target genes were normalized relative to the expression of *β-actin*. Values are means (n = 3) with standard errors represented by vertical bars. Mean values for the same gene with different letters were significantly different (*P* < 0.05).

The expression levels of genes of the inflammatory response in liver and intestine after LPS stimulation are shown in **Figure 8**. In liver, the highest expression levels of *il-1β* and *nf-κb* were recorded in fish fed HFD, and they were significantly down-regulated in fish fed the HFD+B2 diet (**Figure 8A**). The anti-inflammatory cytokine *il-10* was significantly up-regulated in fish fed the diets supplemented with betaine compared to fish fed HFD. There were no significant differences in the hepatic expression levels of *tgfβ-1* or *tnf-α* among treatments. In intestine, the results were similar to those of liver, with highest expression levels of *nf-κb* and pro-inflammatory cytokine *tnf-α* obtained in fish fed HFD, while they were significantly down-regulated in fish fed diets supplemented with betaine (HFD+B2) (**Figure 8B**). However, fish fed dietary betaine supplementation showed significantly higher expression levels of *il-10* and *tgfβ-1* than fish fed HFD. No statistical differences in expression of *il-1β* were found among treatments. In kidney, there were clear trends for dietary betaine to slightly decrease the expression levels of *nf-κb* and *il-1β* mRNA compared with fish fed HFD, but no statistical differences were found (**Figure 8C**). Fish fed diets containing betaine showed reduced relative expression levels of *tnf-α*, whereas expression levels of *il-1β* and *tgfβ-1* were upregulated in fish fed dietary betaine.

## Discussion

Betaine is a common additive in aquafeeds, with diets supplemented with betaine reported to improve growth performance and feed intake in various fish species such as allogynogenetic gibel carp (8), blunt snout bream (11), pike perch (*Sander lucioperca*) (40), common carp (13) and Nile tilapia (*Oreochromis niloticus*) (15). Consistent results were also obtained in the present study, where SGR and FE increased with increasing betaine supplementation compared to fish fed HFD, indicating that 2% dietary betaine resulted in best growth performance and feed utilization.

It has been reported that fat accumulation was associated with impairment of hepatic one-carbon metabolism (41), with lower of SAM content and increased Hcy level (42, 43). As betaine is an essential component of the hepatic methionine-homocysteine cycle and acts as a donor of methyl groups (21), we therefore measured key metabolites of this cycle including SAM, SAH and Hcy. Fish fed HFD showed slightly decreased hepatic SAM content compared to fish fed the control diet and SAM level was increased by dietary betaine supplementation and, although not statistically significant, similar results were found for hepatic SAH concentration. These data are in accordance with previous reports in rat, which demonstrated that dietary betaine could prevent methionine cycle metabolic disorders caused by high fat diets (16, 21, 44). Furthermore, compared to fish fed diets supplemented with betaine, a high content of hepatic Hcy was recorded in fish fed diet HFD, which indicated that the methionine cycle was impacted by HFD. In contrast, hepatic Hcy levels were decreased in fish fed HFD+B1, suggesting that betaine provided the methyl group and, in turn, restored methionine cycle metabolism to normal, as reported previously in mammals (16, 21). This demonstrated that methionine cycle disorders can be associated with HFD-induced hepatic steatosis, and diets supplemented with betaine can restore normal methionine cycle metabolism and, consequently, attenuate liver injury. However, further studies are required in this important area.

High fat diets in fish can readily cause liver injury, with the most obvious being increased lipid deposition and higher tissue lipid contents and, in the present study, the highest hepatic lipid content was observed in fish fed the HFD diet. As expected, fish fed diets supplemented with betaine showed significantly reduced hepatic lipid content in accordance with previous studies, confirming that dietary betaine could reduce hepatic lipid content in fish (8, 11) as in rats (16). Furthermore, hepatic histopathological analysis indicated that fish fed HFD showed clear liver injury including the presence of many large vacuolar lipid droplets. Similar results were reported previously in mammals (16) and fish species (5–7, 45, 46). However, histopathological analyses also revealed that dietary betaine supplementation was beneficial in reducing hepatic lipid accumulation caused by HFD, with fewer and smaller lipid droplets observed, consistent with results reported previously in rats and mice (16, 19, 20, 47, 48). In addition, a previous study in mammals demonstrated that ADP may function in lipid metabolism by increasing fatty acid oxidation in tissues such as liver and muscle (49). In the present study, hepatic ADP contents were measured and, although no statistically differences were found among treatments, fish fed diet supplemented with betaine showed numerically higher ADP content, suggesting that dietary betaine may have the potential to improve fatty acid oxidative catabolism. Overall, the present study indicated that dietary betaine supplementation can reduce fat accumulation and prevent liver damage caused by HFD, confirming that dietary betaine can have beneficial lipid-lowing effects in black seabream fed high fat diets.

Based on the lipid-lowing effects of dietary betaine reported above, the mechanism of betaine in reducing lipid deposition caused by HFD was further studied by investigating some lipid metabolism related pathways. In the present study, as a key lipolytic regulator factor, *pparɑ* expression was increased by dietary betaine, and similar trends were found in downstream gene expression, such as *cpt1a* and *lpl*. Furthermore, in fish fed the diets supplemented with betaine, the expression of *srebp-1*, the main regulatory transcription factor of lipogenic pathways, was down regulated. This was consistent with the results obtained for *fas* and *accɑ*, which are downstream gene targets of *srebp-1*, and in accordance with results reported previously in mammals (21, 47, 50) and fish (8, 11), demonstrating that dietary betaine could reduce lipid deposition by enhancing Pparɑ and its downstream lipolytic gene targets, and by repressing Srebp-1 and its downstream lipogenic gene targets. However, in the present study, both mRNA expression and protein concentration of Pparɑ and Srebp-1 were regulated by dietary betaine. Studies in mammals showed that Sirt1 was a negative modulator of the SREBP-1 lipogenic pathway (28–31) and, furthermore, as a key pathway for the regulation of hepatic lipid metabolism, SIRT1 positively regulated PPARα to reduce hepatic lipid deposition (27). Therefore, Sirt1 has already emerged as an integral component of these metabolic pathways in mammals (51, 52) and, hence, we speculated that Sirt1 might be impacted by dietary betaine supplementation and, consequently, modulate lipid metabolic pathways in black seabream. As predicted, hepatic *sirt1* gene expression level was upregulated in fish fed the diets supplemented with betaine. Moreover, a similar result was found for liver protein expression of Sirt1, which was enhanced by dietary betaine. This is first time that dietary betaine has been reported to operate through modulating Sirt1 to regulate lipid metabolic pathways (Pparɑ and Srebp-1) in a teleost, consequently alleviating lipid metabolism disorders and attenuate hepatic steatosis.

Previously we showed that high fat diets could cause hepatic steatosis and, consequently, induce inflammatory responses in black seabream (5, 6). Likewise, the present study indicated that expression of *nf-kb*, *tnf-α* and *il-1β* were all up-regulated in liver and intestine in fish fed HFD when compared to fish fed the Control, medium fat diet. The nuclear transcription factor *nf-kb* is a vital upstream signaling molecule and, when NF-κB is activated, it transfers into the nucleus and promotes the expression of pro-inflammatory markers, including *tnf-α* and *il-1β* (6, 53, 54). Interestingly, in the current study, the HFD-induced inflammation response could be mitigated by dietary betaine, with fish fed betaine supplementation showing decreased expression of *nf-kb*, *tnf-α* and *il-1β* in liver and intestine. These results were consistent with previous studies in rat and grass carp (*Ctenopharyngodon idella*), which reported that betaine could ameliorate inflammation by suppressing NF-κB activation (19, 25). Furthermore, a previous study in mammals reported that PPARα suppressed expression of proinflammation markers through down-regulation NF-κB by a SIRT1-mediated mechanism (34). In the present study, expression levels of *pparɑ* and *sirtl* were increased, whereas expression of *nf-kb*, *tnf-α* and *il-1β* were decreased by dietary betaine supplementation. To some extent, these results were also demonstrated in large yellow croaker (*Larmichthys crocea*), confirming that the NF-kB p65 subunit can be deacetylated by Sirt1, showing an inflammation-lowering effect (54). Moreover, some studies reported previously that Sirt1 could suppress NF-kB signaling that, in turn, greatly attenuated NF-kB driven inflammation (32, 33). Overall, the present study revealed that dietary betaine supplementation alleviated hepatic steatosis-induced inflammation through Sirt1/Pparɑ signaling pathway-mediated suppression of NF-kB and, consequently, attenuated NF-kB driven inflammation response in black seabream.

In order to further verify that dietary betaine supplementation had the beneficial effect of alleviating inflammation, an LPS challenge experiment was conducted to promote a strong inflammatory response. A previous study confirmed that, in teleosts, inflammatory challenges *in vivo* and *in vitro* are able to induce expression of various pro-inflammatory factor genes with rapid kinetics (55). Furthermore, it was reported that LPS could rapidly stimulate the expression of *il-1β*, and *tnf-α* (56). In the present study 24 h after LPS injection, fish fed HFD showed highest expression levels of *nf-kb*, *il-1β* and *tnf-α* in liver, intestine and kidney, similar to results found in other studies (4, 6, 57, 58). However, expression of *nf-kb*, *il-1β* and *tnf-α* were decreased by dietary betaine supplementation. On the contrary, *tgfβ-1* and *il-10* expression levels were up-regulated by dietary betaine supplementation in all tested tissues, except for *tgfβ-1* in intestine and *il-10* in kidney. These results were consistent with the previously reported studies. Therefore, the LPS injection experiment generally confirmed that dietary betaine supplementation had an inflammation-lowing effect by regulating expression of inflammatory cytokines.

## Conclusion

**Figure 9:**
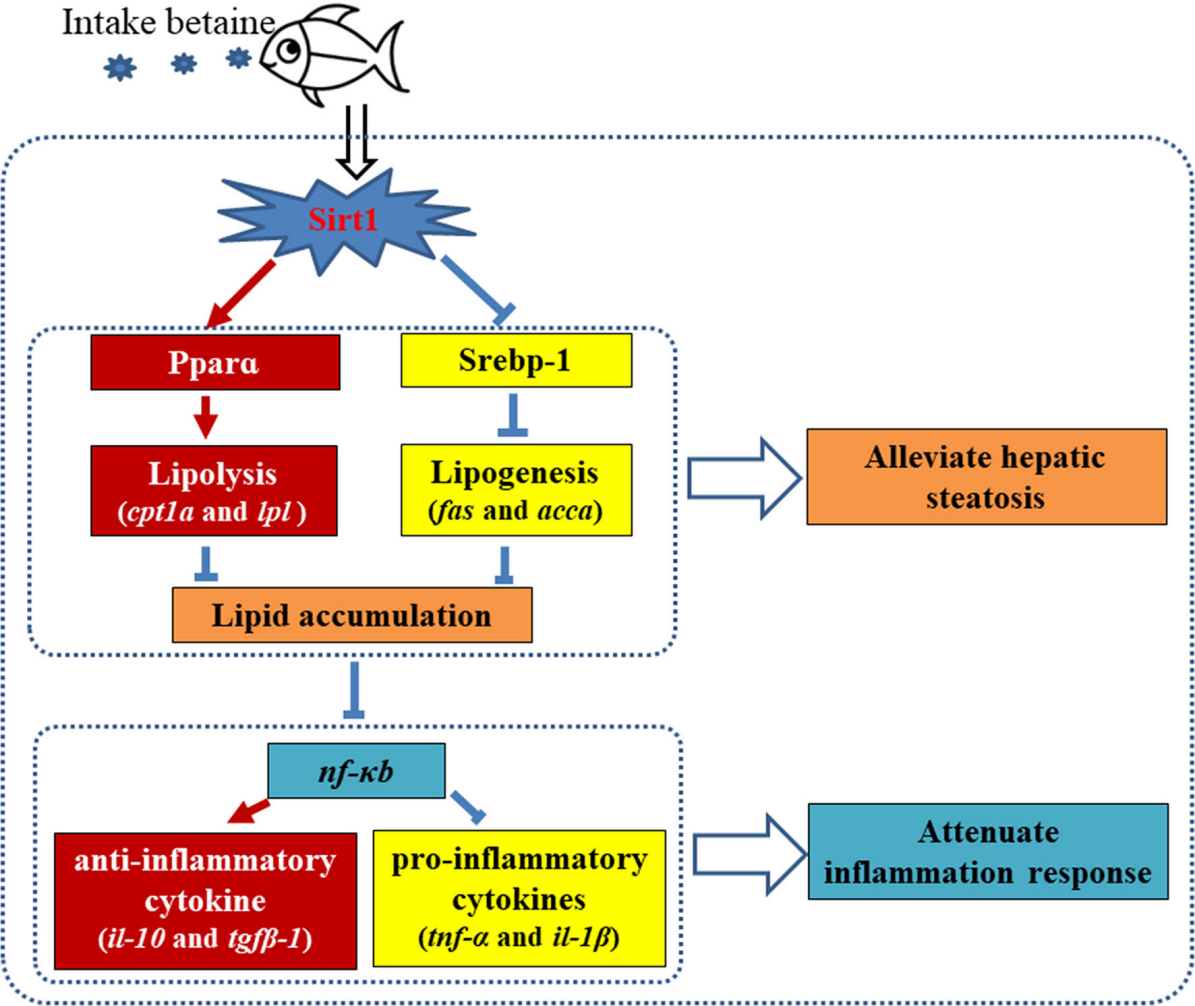
Proposed mechanism of dietary betaine supplementation on alleviating hepatic steatosis and attenuating inflammation responses caused by high fat diets in black seabream (*Acanthopagrus schlegelii*). Red arrows represent promotion/up-regulation, blue symbols present repression/downregulation.

In conclusion, the present study has provided further insight into the mechanisms of betaine attenuation of high-fat diet-induced hepatic steatosis and inflammatory responses through modulating the Sirt1/Srebp-1/Pparɑ pathway. The current study revealed that dietary betaine supplementation could through promote the mRNA expression and protein levels of Sirt1 to modulate the main regulatory transcription factors of lipid metabolism including Srebp-1 and Pparɑ, demonstrating that Sirt1 mediated the effects of betaine by repressing lipogenic pathways and promoting lipolytic pathways (**Figure 9**). The present study also suggested that HFD-induced hepatic steatosis can disturb the homeostasis of the methionine cycle, and that dietary betaine may restore methionine cycle metabolism to a normal condition and, consequently, attenuate hepatic lipid metabolism disorders. Furthermore, our findings revealed that dietary betaine supplementation alleviated hepatic steatosis-induced inflammation through Sirt1/PPARɑ signaling pathway mediated suppression of NF-kB and, consequently, attenuated NF-kB driven inflammation response in black seabream.

## Data Availability Statement

The datasets presented in this study can be found in online repositories. The names of the repository/repositories and accession number(s) can be found in the article/supplementary material.

## Ethics Statement

Animal experimentation within the present study was conducted in accordance with the Animal Research Institute Committee guidelines of Ningbo University, China and approved by the Committee of Animal Research Institute, Ningbo University, China.

## Author Contributions

QZ, MJ, DT, and YS: Conceptualization, Methodology, Validation. YS, TP, TZ, and XL: Formal analysis. LJ and FX: Resources. MJ, YS, and TP: Writing - Original Draft. MJ, DT, and MB: Writing- Reviewing and Editing. All authors contributed to the article and approved the submitted version.

## Funding

This research was supported by the National Natural Science Foundation of China (31802303), National Key R & D Program of China (2018YFD0900400), Scientific Research Foundation of Ningbo University (XYL20007), Fundamental Research Funds for the Provincial Universities of Zhejiang (SJLY2021007), Natural Science Foundation of Ningbo (2018A610343), Key Research Program of Zhejiang Province of China (2018C02037), Industrial Chain Collaborative Innovation Project of the Demonstration Work on Innovative Development of the Marine Economy of the State Oceanic Administration (NBHY-2017-S2), Zhejiang Aquaculture Nutrition & Feed Technology Service Team (ZJANFTST2017-2), the Open Fund of Zhejiang Provincial Top Key Discipline of Aquaculture in Ningbo University and K. C. Wong Magna Fund in Ningbo University.

## Conflict of Interest

The authors declare that the research was conducted in the absence of any commercial or financial relationships that could be construed as a potential conflict of interest.

## Abbreviations

ADP, hepatic adiponectin; CHOL, cholesterol; ERS, Endoplasmic reticulum stress; FE, Feed efficiency; Hcy, Homocysteine; HFD, High-fat-diet; HFD+B1, HFD with betaine supplement (10g/kg dry diet); HFD+B2, HFD with betaine supplement (20g/kg dry diet); LPS, lipopolysaccharide; LXR, Liver X receptor; SAH, S-adenosylhomocysteine; SAM, S-adenosylmethionine; SGR, Specific growth ratio; Sirt1, Silent information regulator 1; TG, triacylglycerol; *accα*, Acetyl-CoA carboxylase alpha; *cpt1a*, Carnitine palmitoyltransferase 1A; *fas*, Fatty acid synthase; *il-10*, Interleukin-10; *il-1β*, Interleukin 1 beta; *lpl*, Lipoprotein lipase; *nf-κb*, Nuclear factor-kappa B; *ppar*α, peroxisome proliferators-activated receptor alpha; *srebp-1*, Sterol regulator element-binding protein-1; *tgfβ-1*, Transforming growth factor beta-1; *tnf-α*, Tumor necrosis factor alpha.
